# CCN2/Connective Tissue Growth Factor Is Essential for Pericyte Adhesion and Endothelial Basement Membrane Formation during Angiogenesis

**DOI:** 10.1371/journal.pone.0030562

**Published:** 2012-02-20

**Authors:** Faith Hall-Glenn, R. Andrea De Young, Bau-Lin Huang, Ben van Handel, Jennifer J. Hofmann, Tom T. Chen, Aaron Choi, Jessica R. Ong, Paul D. Benya, Hanna Mikkola, M. Luisa Iruela-Arispe, Karen M. Lyons

**Affiliations:** 1 Department of Molecular, Cell and Developmental Biology, University of California Los Angeles, Los Angeles, California, United States of America; 2 Department of Orthopaedic Surgery, University of California Los Angeles, Los Angeles, California, United States of America; 3 Department of Oral Biology, University of California Los Angeles, Los Angeles, California, United States of America; 4 Molecular Biology Institute, University of California Los Angeles, Los Angeles, California, United States of America; 5 Jonsson Comprehensive Cancer Center, University of California Los Angeles, Los Angeles, California, United States of America; University of Bristol, United Kingdom

## Abstract

CCN2/Connective Tissue Growth Factor (CTGF) is a matricellular protein that regulates cell adhesion, migration, and survival. CCN2 is best known for its ability to promote fibrosis by mediating the ability of transforming growth factor β (TGFβ) to induce excess extracellular matrix production. In addition to its role in pathological processes, CCN2 is required for chondrogenesis. CCN2 is also highly expressed during development in endothelial cells, suggesting a role in angiogenesis. The potential role of CCN2 in angiogenesis is unclear, however, as both pro- and anti-angiogenic effects have been reported. Here, through analysis of *Ccn2*-deficient mice, we show that CCN2 is required for stable association and retention of pericytes by endothelial cells. PDGF signaling and the establishment of the endothelial basement membrane are required for pericytes recruitment and retention. CCN2 induced PDGF-B expression in endothelial cells, and potentiated PDGF-B-mediated Akt signaling in mural (vascular smooth muscle/pericyte) cells. In addition, CCN2 induced the production of endothelial basement membrane components *in vitro*, and was required for their expression *in vivo*. Overall, these results highlight CCN2 as an essential mediator of vascular remodeling by regulating endothelial-pericyte interactions. Although most studies of CCN2 function have focused on effects of CCN2 overexpression on the interstitial extracellular matrix, the results presented here show that CCN2 is required for the normal production of vascular basement membranes.

## Introduction

CCN2, also known as connective tissue growth factor, is a member of the CCN (CCN1-6) family of matricellular proteins. CCN family members are cysteine-rich and contain an N-terminal secretory peptide, followed by four multi-functional domains that interact with a diverse array of binding partners [1], [2]. Proteins that interact with CCN2 through recognition of these domains include integrins, low-density lipoprotein receptor-related proteins (LRPs), growth factors, and extracellular matrix (ECM) components. The first domain shares homology to insulin-like growth factor binding proteins (IGFBPs), but has very low affinity for IGF [3]. The second domain encodes a von Willebrand type C (VWC) repeat. This motif mediates CCN2 interactions with growth factors such as bone morphogenetic proteins (BMPs) and transforming growth factor β (TGFβ) [4]. The third domain is a type-1 thrombospondin (TSP) repeat, known to mediate the ability of CCN2 to bind to ECM proteins, matrix metalloproteinases (MMPs) and integrin α6β1 [5], [6] The final C-terminal (CT) motif contains a cysteine knot similar to those present in many growth factors, including members of the TGFβ superfamily, platelet derived growth factor (PDGF), and nerve growth factor (NGF). This motif mediates interactions with integrins αvβ3, α5β1, and α6β1 [7]–[13].

CCN2 was originally isolated from human umbilical vein endothelial cells (HUVECs) [14]. *In situ* hybridization and immunohistochemical studies demonstrated that CCN2 is expressed predominantly in endothelial cells in embryonic and adult vasculature [15]–[18]. The physiological role of CCN2 in angiogenesis is unclear, however, as it appears to have both pro- and anti-angiogenic activities *in vitro*. For example, CCN2 induces corneal angiogenesis, and anti-CCN2 antibodies block angiogenesis in the chick chorioallantoic membrane assay [19], [20]. On the other hand, anti-angiogenic activities have been reported; although *Ccn2* expression is induced by VEGF [21], CCN2 binds to and sequesters VEGF in an inactive form [5], and combined administration of CCN2 and VEGF inhibits VEGF-induced angiogenesis [22]. The role of CCN2 in angiogenesis *in vivo* is unknown.

The majority of studies have focused on the role of CCN2 as a stimulator of excess ECM production in the context of pathological fibrosis [23]. CCN2 is overexpressed in all fibrotic conditions described to date, and depending on the tissue involved, induces collagen type I deposition and increased susceptibility to injury [24]. Conversely, the loss of CCN2 in fibroblasts results in decreased collagen deposition and resistance to chemically induced skin fibrosis [25], [26]. In addition to its role as a mediator of fibrosis, CCN2 is required for ECM production in cartilage [27]. *Ccn2* knockout mice survive in Mendelian ratios throughout gestation, but die within minutes of birth. They exhibit severe chondrodysplasia as a result of decreased collagen type II and aggrecan expression by chondrocytes *in vivo* and *in vitro* [27], [28]. CCN2 regulates cell survival, adhesion, migration, and ECM production in multiple cell types by regulating integrin expression and activation [13]. In *Ccn2* mutant chondrocytes, integrin α5β1 expression and downstream focal adhesion kinase (FAK) and extracellular signal-related kinase (ERK1/2) signaling are decreased, indicating that CCN2 regulates ECM production through integrins [28].

In endothelial cells, CCN2 mediates adhesion, migration and survival through binding to integrin αvβ3 [7]. CCN2 is also a ligand for α5β1 and α6β1 [13], and these integrins are required for endothelial basement membrane formation and vessel stabilization *in vitro* [29]. Taken together, these studies implicate CCN2 as an important regulator of cellular adhesion and ECM production during angiogenesis, but do not address its role *in vivo*. As CCN2 is the major mediator of excess ECM production during fibrosis, and has also been implicated in tumor angiogenesis [30], it is important to understand its function in normal tissues. Therefore, the function of CCN2 in angiogenesis was investigated through analysis of *Ccn2* mutant mice.

## Results

### CCN2 is expressed in the developing vasculature

**Figure 1:**
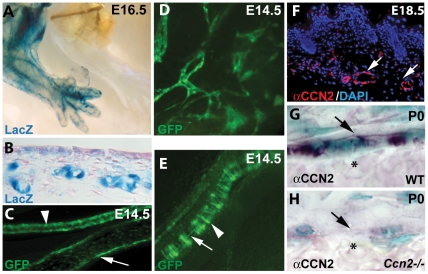
Expression of *Ccn2* in developing vasculature. (A) β-galactosidase activity in *Ccn2-lacZ* transgenic mice reveals *Ccn2* promoter activity throughout the vasculature in E16.5 embryos. (B) *Ccn2-lacZ* expression in dermal microvessels at E13.5. (C–E) EGFP fluorescence in CCN2-EGFP BAC transgenic mice demonstrates CCN2 expression in the endothelium of arterial elements (C and E), venous elements (C), and developing capillary networks (D). Arrowheads in (C) and (E) demarcate arterial element. Arrow in (C) identifies endothelial cells of a venous element. Arrowhead in (E) highlights EGFP expression in mural cells in the arterial element. Arrow in (E) highlights expression in endothelial cells in the arterial element. (F) Immunofluorescence and (G,H) immunohistochemical staining with an αCCN2 antibody on paraffin sections through dermis, demonstrating CCN2 expression in endothelial cells. Arrows in (F) highlight endothelial cells in E18.5 microvasculature. Specificity of the αCCN2 antibody is demonstrated by the absence of reactivity in the *Ccn2−/−* section (H). Arrows in (G) and (H) demarcate abluminal surface of the endothelium. Asterisks in (G) and (H) identify blood cells within the vessels. αCCN2 staining in (G) shows punctate intracellular expression, presumably with the Golgi, in addition to the surface expression marked by the arrow.

Using transgenic mice in which lacZ expression is driven by the 4 kb proximal *Ccn2* promoter [31], CCN2 expression was seen throughout the vasculature and microvasculature at E16.5 (Figure 1A). Expression was observed in large vessels, arterioles and capillaries at all stages examined (E13.5-P0). CCN2 was detected as early as E13.5 in developing dermal microvasculature (Figure 1B), where lacZ is present in large and small caliber vessels (Figure 1A,B). Similar results were seen using bacterial artificial chromosome (BAC) transgenic mice expressing enhanced green fluorescent protein (EGFP) under the control of the *Ccn2* locus (CCN2-EGFP) [32]. This analysis revealed *Ccn2* expression in endothelium of arterial and venous elements, and in capillaries. In large arteries, CCN2-EGFP was expressed in both endothelial and vascular smooth muscle cells (vSMCs) (Figure 1C,E). CCN2 was also expressed in developing capillary networks (Figure 1D). Endothelial-specific expression in microvasculature was also shown by immunostaining for CCN2 (Figure 1F–H). Specificity of the antibody was confirmed by the absence of staining sections from *Ccn2* mutants (Figure 1H). Punctate intracellular staining was observed, most likely within the Golgi and in secretory vesicles, as reported previously [33]. Cell-associated expression was also seen on the abluminal surface of the endothelium (Figure 1G). Co-immunostaining with the endothelial-specific marker PECAM (CD31) revealed CCN2 expression in endothelial cells and in mural cells (Figure S1A). Thus, *Ccn2* is expressed in both endothelial and mural cells in blood vessels and capillaries during development.

### *Ccn2* mutant mice exhibit vascular defects

**Figure 2:**
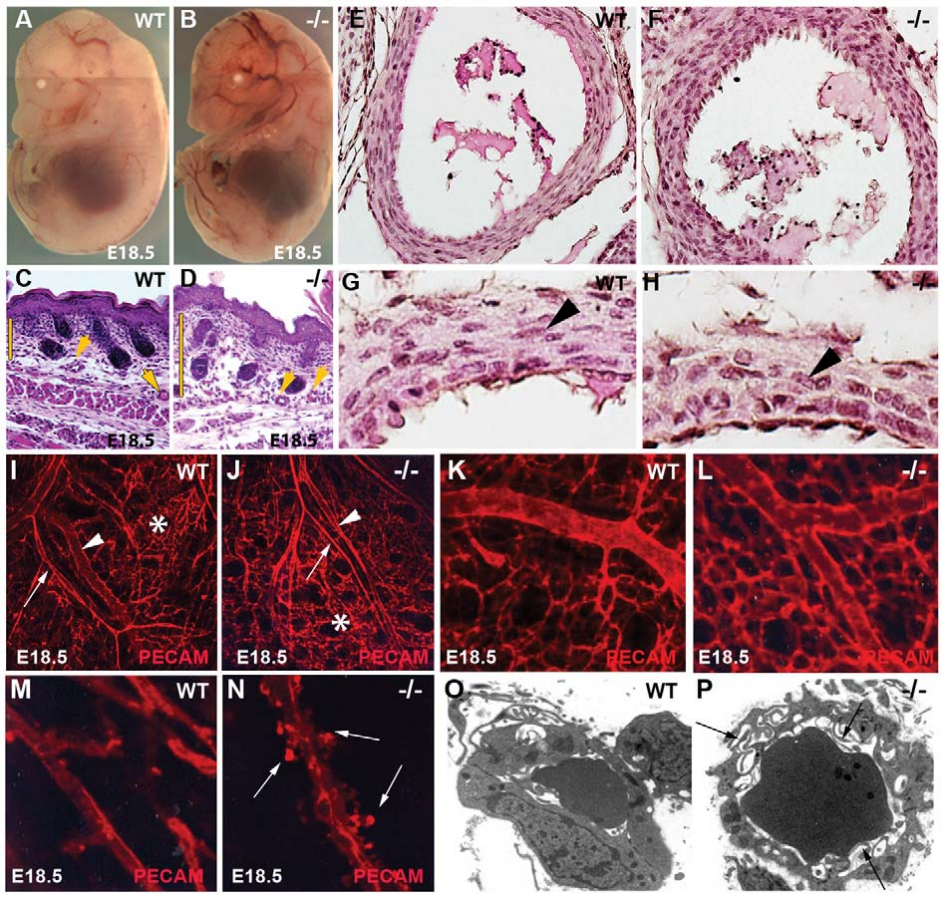
Vascular abnormalities in *Ccn2* mutant embryos. (A) E18.5 WT and (B) *Ccn2−/−* littermate, showing vessel dilation throughout the mutant embryo. (C, D) H&E-stained paraffin sections through the lumbar dorsal dermis of (C) E18.5 WT and (D) *Ccn2−/−* littermate. Arrowheads point to vessels. Bars highlight the enlarged distance between the hypodermal and epidermal layers in the mutant, indicative of local edema. (E,F) Hematoxylin and eosin-stained sections through E16.5 WT (E) and *Ccn2−/−* (F) descending aorta at thoracic level. Smooth muscle cells in the tunica media are spindle-shaped and arranged in layers in the WT embryo, but are more cuboidal and disorganized in the *Ccn2−/−* littermate. (G,H) Higher magnification images through aorta at lumbar level in E16.5 (G) WT and (H) *Ccn2−/−* littermate showing spindle-shaped smooth muscle cells (arrowheads) in WT that have a cuboidal shape in the mutant. (I,J) Confocal images of PECAM-stained dorsal dermal vasculature in (I) WT and (J) *Ccn2−/−* littermates. Arrows demarcate arterial elements; arrowheads demarcate venous elements; asterisks identify capillary beds. (K,L) Higher magnification confocal images of (K) WT and (L) *Ccn2−/−* dorsal dermal capillary beds, showing increased capillary density in the mutant. (M,N) High magnification confocal image of (M) WT and (N) *Ccn2−/−* dorsal dermal capillaries, showing numerous abluminal protrusions (arrows in (N)) on the mutant capillary. (O,P) Electron micrographs of newborn (P0) (O) WT and (P) *Ccn2−/−* dermal capillaries, showing abluminal and luminal (arrows in (P)) protrusions.

*Ccn2* mutant mice exhibit perinatal lethality due to a severe chondrodysplasia [27]. CCN2 expression in developing blood vessels raised the possibility of an additional role in vascular development. *Ccn2−/−* embryos were examined to investigate this possibility. No overt differences between *Ccn2* mutants and WT littermates were apparent during the initial formation of the vasculature from E9.5–E13.5 (data not shown). Moreover, placentas were normal in appearance, weight, and vascularity throughout development (Figure S1B,C, and data not shown). However, beginning at E14.5, minor enlargement of vessels was observed in mutants (Figure S1D,E), which became more pronounced at later stages (Figure 2A,B). Local edema was seen in E18.5 mutant dermis (Figure 2C,D). Immunofluorescence analysis of the vSMC marker smooth muscle actin (SMA) and PECAM (CD-31) did not reveal obvious evidence that SMC coverage of large vessels was affected in mutants (Figure S1F–I). However, comparison of hematoxylin and eosin-stained sections of the aorta at thoracic and lumbar levels from E16.5 embryos showed defects in the organization of the tunica media (Fig. 2E–H). In WT embryos, SMCs had a spindle-like morphology and were circumferentially oriented around the vessel lumen in distinct layers (Figure 2E,G). In mutants, SMCs failed to adopt this spindle-like morphology, were more heterogeneous in size, and were not organized into distinct layers (Figure 2F,H). The large vessel phenotype will be reported in more detail elsewhere. Here we focus on the microvascular phenotype.

Morphological examination (Figure S1J,K) revealed that arterial-venous identity appeared to be maintained in mutants (see also Figure S1H,I). Ephrin B2 (expressed on arterial elements) and EphB4 (preferentially expressed on veins) staining demonstrated no defects in arterial-venous identity (Figure S1L,M, and data not shown). However, inspection of E18.5 dermal microvasculature revealed evidence of defective remodeling in *Ccn2* mutants. Consistent with a defect in remodeling, vessel density was increased in *Ccn2* mutants (Figure 2I–L and Figure S2A–C). Moreover, mutant capillaries had multiple protrusions along their surfaces (Figure 2M,N). Electron microscopy revealed numerous luminal and abluminal protrusions in mutant capillaries, consistent with the confocal analysis (Figure 2O,P).

### CCN2 mutants exhibit defects in vascular remodeling

PCNA labeling and TUNEL analyses were performed to assess whether defects in proliferation and/or survival might contribute to the microvascular abnormalities in *Ccn2* mutants. No differences were detected in mutants in comparison to WT littermates (Figure S2D–G). During vascular remodeling, immature vascular beds become less dense, arterioles become smaller in diameter than venules, and pericytes form stable associations with endothelial tubes [34]. Angiopoetin 1 (Ang1) is required for stabilizing endothelial-pericyte interactions and is expressed primarily by mural cells [35]. *Ang1* mRNA levels were diminished in *Ccn2−/−* skin (Figure S2H). No differences were detected in levels of expression of *Tie2*, the endothelial-specific receptor for Ang1 (data not shown). However, levels of the mRNA encoding the bio-active VEGF isoform 164 were elevated in mutants (Figure S2I). Versican is the principal chondroitin sulfate proteoglycan in blood vessels and exists in at least four isoforms, V0, V1, V2, and V3 [36]. Embryonic endothelial cells express more V0 than other isoforms, and V0 expression declines during vascular maturation [37]. No differences were seen in levels of versican *V1* in *Ccn2* mutants and WT littermates (Figure S2J); however, *Ccn2* mutants exhibited increased levels of *V0* (Figure S2K). Therefore, the loss of *Ccn2* leads to diminished expression of vessel maturation marker *Ang1* and elevated expression of markers of immature vasculature, indicative of a potential defect in vascular remodeling.

**Figure 3:**
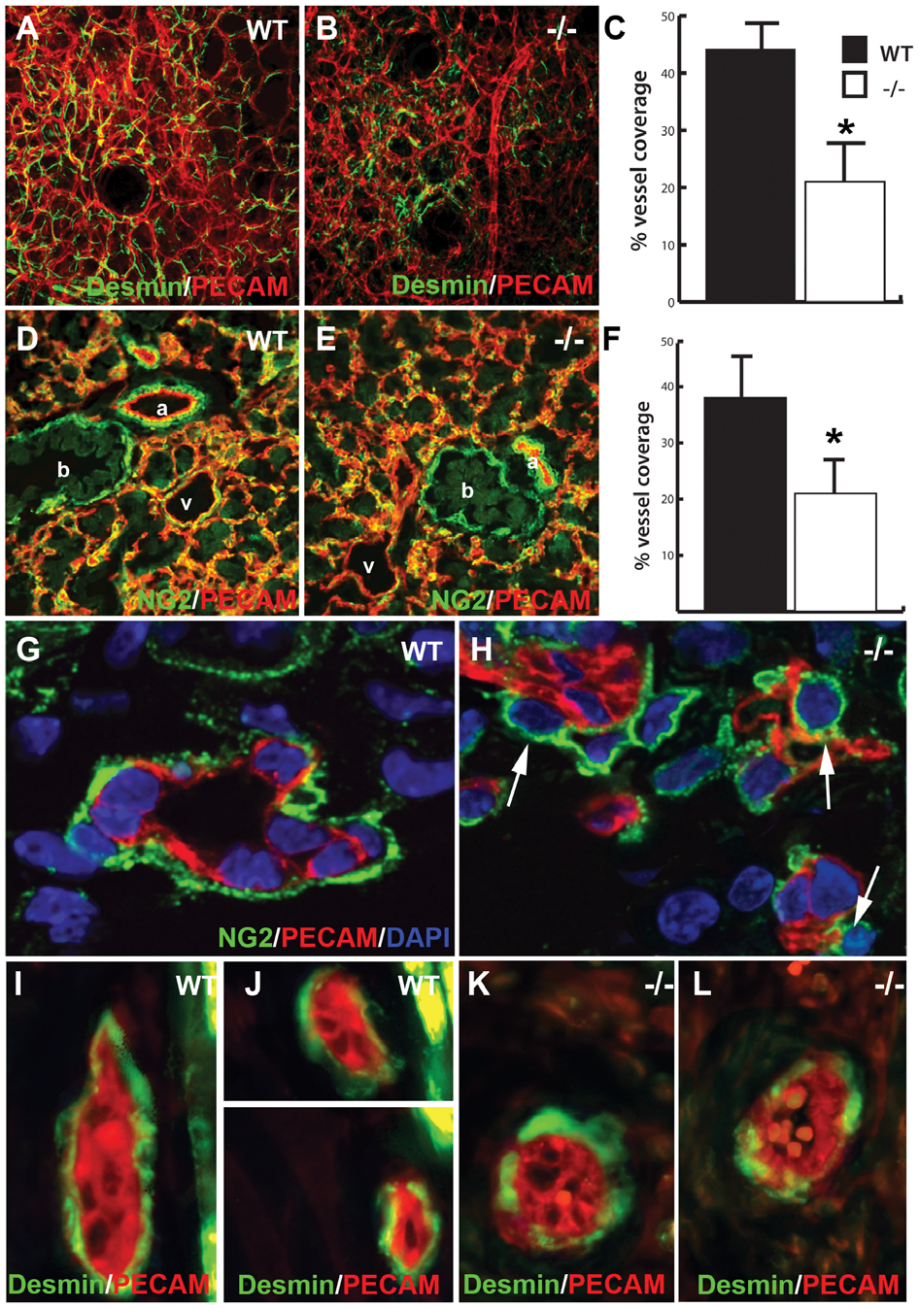
Defective endothelial-pericyte interactions in *Ccn2* mutants. (A, B) Co-immunofluorescence staining for desmin and PECAM in E18.5 dermis from (A) WT and (B) *Ccn2−/−* mice analyzed by confocal microscopy. (C) Quantification of vessel coverage by pericytes in E18.5 dermis; asterisk, p<0.05. (D, E) Co-immunofluorescence staining for NG2 and PECAM in E16.5 lung from (D) WT and (E) *Ccn2−/−* mice analyzed by confocal microscopy. (F) Quantification of vessel coverage by pericytes in E16.5 lung; asterisk, p<0.05. (G,H) Confocal analysis of NG2 and PECAM immunostaining in (G) WT and (H) *Ccn2−/−* E16.5 dermis. Pericytes are elongated around the microvessel in (G), whereas in mutants (H), pericytes (arrows) are associated with the endothelium, but are rounder, and fewer of them have elongated along the endothelial surface. (I–L) Confocal sections through E16.5 dermis analyzed for desmin (green) and PECAM (red) immunofluorescence. (I,J) WT desmin positive pericytes appear elongated and cover most of the surface of the microvessels. (K.L) *Ccn2−/−* desmin-positive pericytes have a rounder appearance and show less extensive coverage of the surface of the endothelium.

The vascular phenotype in *Ccn2* mutants bears some resemblance to mice lacking platelet-derived growth factor-B (PDGF-B) or its receptor, PDGFRβ [38], [39]. In particular, defective pericyte recruitment is seen in these mice. Therefore, we examined pericyte recruitment in *Ccn2* mutants. Pericytes, which express NG2 and desmin, become associated with small diameter vessels during vessel maturation [40]. Consistent with the gene expression analysis described above, confocal analysis of desmin expression revealed incomplete coverage of microvessels by pericytes in the dermis of *Ccn2* mutants at E16.5 and E18.5 (Figure 3A–C; data not shown). Similar results were seen for NG2 expression in the lung liver, and brain microvasculature (Figure 3D–F, and data not shown). Thus, the loss of CCN2 affects the microvasculature in multiple tissues. Flow cytometric analysis of lung, liver, and brain samples from E16.5 embryos for cells negative for the endothelial cell marker PECAM, but expressing the pericyte markers NG2 and PDGFRβ [41] revealed normal numbers of endothelial cells and pericytes in *Ccn2* mutants (Figure S3, and data not shown). This suggests that the reduced pericyte coverage in *Ccn2* mutants is not caused by a decrease in pericyte number or migration, but possibly by defects in the ability of pericytes to make stable associations and elongate along endothelial cells in *Ccn2* mutant mice.

Confocal analysis of E16.5 dermal and lung microvasculature co-stained with NG2, desmin, and PECAM supports this possibility. NG2 staining demonstrated that pericytes associated with WT vessels were in close contact with the capillary endothelium and appeared elongated along the endothelial surface (Figure 3G). In contrast, pericytes associated with capillaries in mutants were more rounded and exhibited less elongation (Figure 3H). Immunostaining with desmin also suggested a defect in pericyte association with endothelial cells in mutants. In WT capillaries, pericytes were elongated and covered the surface of endothelial tubes (Figure 3I,J and Figure S4A,B). In contrast, pericytes on mutant capillaries were rarely elongated, and vessel coverage was incomplete (Figure 3K,L and Figure S4C,D). Taken together, these findings indicate that the ability of pericytes to form stable associations with microvascular endothelium is defective in *Ccn2* mutants.

### CCN2 potentiates PDGF signaling in vascular cells

PDGF-B, produced by endothelial cells, and its receptor, PDGFRβ expressed in pericytes, are required for pericyte recruitment to nascent vessels [38]. CCN2 was originally identified as a protein that competes with PDGF-B for binding to NIH 3T3 cells, leading to the suggestion that CCN2 binds to PDGF receptors [42]. However, subsequent studies using a C-terminal isoform of CCN2 showed no interaction between CCN2 and PDGF receptors [43]. We tested whether full-length CCN2 interacts with PDGF-B or its receptor through co-immunoprecipitation and found no evidence for a direct physical interaction (Figure S5, Methods S1). These findings suggest that CCN2 does not influence PDGF signaling by interacting directly with PDGF-B or PDGFRβ.

**Figure 4:**
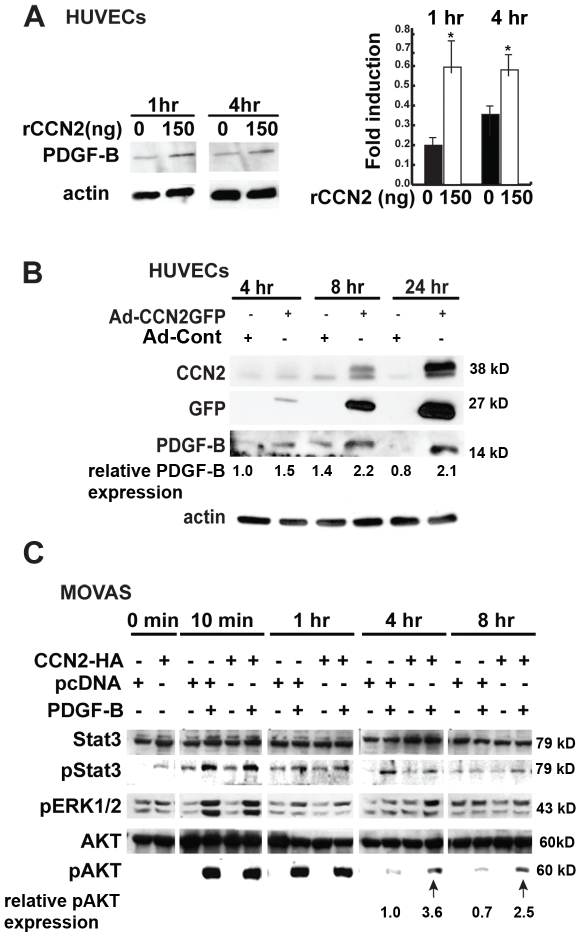
CCN2 potentiates PDGF-B signaling. (A) rCCN2 induces PDGF-B expression in HUVEC cells. Right panel, representative Western blot. Left panel, Quantification of relative expression levels of PDGF-B in cells treated with or without rCCN2 from three separate experiments. *, p<0.02. (B) Adenovirally expressed CCN2 induces PDGF-B expression in HUVECs compared to transfection with an empty adenoviral control. The extent of PDGF-B induction correlated with levels of CCN2 expression. As reported previously, a higher molecular weight isoform of CCN2, presumably a result of post-translational modification [1], is detected 4 and 8 hours post-infection. Relative level of PDGF-B expression was assessed using ImageJ software. The experiment was repeated three times, with similar results each time. The induction of PDGF-B in the presence of CCN2 was statistically significant for each time point; p<0.05. A representative Western blot is shown. (C) Effects of rPDGF-B, and/or pcDNA3-CCN2-HA expression on activation of PDGF pathways in MOVAS cells. PDGF-B stimulated activation of Stat3, ERK, and Akt, whereas CCN2-HA on its own had no effect. However, combined treatment with PDGF-B and CCN2-HA led to prolonged Akt activation (arrows). Relative levels of pAKT expression were assessed using ImageJ software. All experiments were performed in triplicate and repeated three times, with similar results each time. The increase in pAKT levels in the presence of CCN2 was statistically significant at each time point; p<0.05. A representative Western blot is shown.

Next, we investigated whether CCN2 could induce PDGF-B expression in endothelial cells. Recombinant CCN2 (rCCN2) induced PDGF-B protein expression in human umbilical vein endothelial cells (HUVECs) at 1 and 4 hours of stimulation (Figure 4A). This was confirmed using HUVECs transfected with a CCN2-GFP adenovirus (adCCN2GFP). AdCCN2GFP-transfected cells induced PDGF-B protein expression at all time points tested, and the level of PDGF-B induction correlated with levels of CCN2 expression (Figure 4B). Given that CCN2 induces PDGF-B expression in endothelial cells, the potential effects of CCN2 on PDGF signaling pathways in mural cells, which express PDGFRβ, were investigated. CCN2 on its own did not activate Stat3, ERK1/2, or AKT, whereas PDGF activated all of these pathways. Furthermore, CCN2 had no effect on PDGF-B-induced ERK1/2 or Stat3 activation, but Akt activation was elevated and prolonged upon treatment with PDGF and CCN2 (Figure 4C). Thus CCN2 can potentiate PDGF signaling between endothelial cells and mural cells.

### Components of the endothelial basement membrane are compromised in *Ccn2* mutants

Decreased expression of PDGF-B and reduced PDGF signaling are unlikely to be the entire basis for the *Ccn2* mutant phenotype because endothelial-specific loss of PDGF-B is compatible with survival, and mice having as much as a 90% decrease in pericyte number survive as adults [44]. The basement membrane is essential for coordinating key signaling events that stabilize the vasculature during angiogenesis [45]. The expression of fibronectin (FN) by endothelial cells is an early event in vascular basement membrane formation [46]. The provisional fibronectin matrix provides organizational signals to endothelial cells, and establishes a framework for the incorporation of permanent basement membrane components such as collagen type IV [29], [46], [47]. Defects in basement membrane formation lead to severe defects in angiogenesis [48]–[51]. Because overexpression of CCN2 leads to thickening of glomerular and retinal capillary basement membranes in diabetic mice [52], [53], we investigated whether CCN2 is required for the formation of endothelial basement membranes during development.

**Figure 5:**
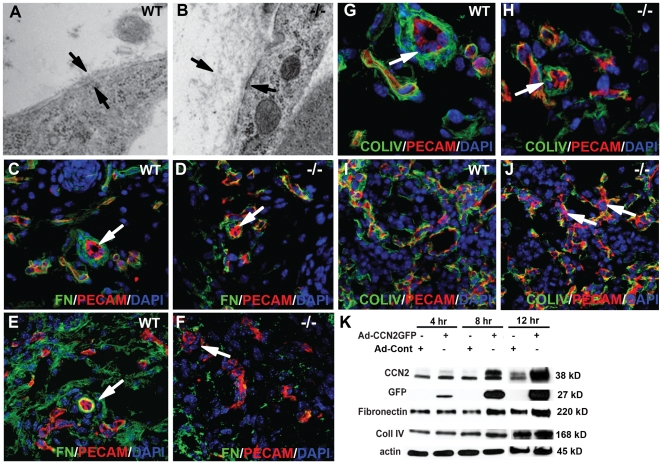
Endothelial basement membrane defects in *Ccn2* mutants. Electron microcopic images of endothelial basement membranes in dermal capillaries of E16.5 (A) WT and (B) *Ccn2−/−* littermates. Arrows demarcate the plasma membrane (bottom arrow) and top of the interstitial matrix (top arrow). (C,D) Confocal images of dermis of E16.5 WT (C) and *Ccn2−/−* (D) mice analyzed by immunofluorescence for fibronectin (FN) and PECAM. Arrows identify an arteriole. The arteriole in (C) is surrounded by several layers of FN. The arteriole in (D) is incompletely invested with FN. (E,F), Lower magnification confocal images through (E) WT and (F) *Ccn2−/−* E16.5 dermis, illustrating less fibronectin throughout the dermis in mutants. (G,H) Confocal images of dermis of E16.5 (G) WT and (H) *Ccn2−/−* mice analyzed by immunofluorescence for ColIV (Col4α2) and PECAM. Arrows identify an arteriole. ColIV coverage of the mutant vasculature is incomplete. (I,J) Confocal images of lungs of E16.5 (I) WT and (J) *Ccn2−/−* mice analyzed by immunofluorescence for ColIV and PECAM. Most of the vascular elements in the WT lung are surrounded by ColIV. Coverage is incomplete in the *Ccn2* mutant lung. Arrows in (J) identify vessels lacking coverage by ColIV. (K) CCN2 induces expression of FN and ColIV in HUVECS. HUVECs were infected with Ad-CCN2-GFP or Ad-control. Lysates were collected at the indicated time points post-infection. Levels of FN are elevated 8 hours after infection, concomitant with accumulation of CCN2. There appeared to be an increase in FN levels at 12 hours in the presence of CCN2 in the blot shown, but this was not seen in every experiment and the result did not reach statistical significance at this time point. Similarly, there was a trend towards increased expression of Col IV at 12 hr, but this increase did not reach statistical significance (p = 0.065). The experiment was repeated three times. A representative blot is shown. Quantification of levels of FN and Col IV are shown in Figure S6.

Electron microscopy provided evidence for defects in microvascular endothelial basement membrane assembly in *Ccn2* mutants. In WT microvessels, the interstitial matrix was compact and localized near the surface of the plasma membrane (Figure 5A). It was more diffuse in mutants (Figure 5B). Therefore, expression of FN and Col4α2 was investigated through confocal analysis. FN expression and association with vessels is significantly decreased in E16.5 *Ccn2* mutant skin and lung vasculature (Figure 5C–F, and data not shown). Collagen type IV expression was also diminished and discontinuous in vascular basement membranes in mutants (Figure 5G–J). Western blot analysis of Ad-CCN2GFP-transfected cells demonstrated that CCN2 induced expression of FN in HUVECs compared to empty vector-transfected controls (Figures 5K and S6). CCN2 had no apparent effect on Col4α2 expression (Figures 5K and S6).

## Discussion

Endothelial cells proliferate and migrate toward the sources of angiogenic signals during development. Upon removal of the angiogenic trigger, a switch to a maturation phase occurs, involving cessation of cell proliferation and migration, followed by the recruitment of mural cells to the vessels, and deposition of the basement membrane. Although the importance of the basement membrane in vascular maturation is widely accepted, the roles of specific ECM components have been difficult to ascertain, especially *in vivo* [45]. Here we show that the matricellular protein CCN2 is a crucial regulator of vascular remodeling.

The results reported here suggest that CCN2 is required for pericyte recruitment in part by potentiating PDGF signaling. We have shown that CCN2 induces expression of PDGF-B in endothelial cells. In turn, CCN2 is induced in pericytes in response to serum or TGFβ [54]. Thus, PDGF and CCN2 appear to be components of a positive feedback loop that operates between endothelial cells and pericytes.

In addition to regulating levels of PDGF-B expression, CCN2 potentiates Akt activation by PDGF-B in vSMCs. Our findings extend previous studies [42] that indicate CCN2 does not interact directly with PDGF-B or PDGFRβ in vascular cells. Thus, CCN2 most likely potentiates the ability of PDGF-B to activate PDGFRβ in mural cells through indirect mechanisms. One of the most plausible of these involves interactions between CCN2 and integrin αvβ3. This integrin is expressed in endothelial cells and pericytes [55], [56]. CCN2 binds to integrin αvβ3 to promote endothelial cell migration and proliferation [9]. Moreover, αvβ3 associates with and potentiates signaling through PDGFRβ [55]. Although our *in vivo* studies cannot address the physiological consequences of altered Akt signaling to the *Ccn2−/−* vascular phenotype, the *Ccn2−/−* phenotype is consistent with the possibility that reduced activation of Akt makes a contribution; *Akt1−/−* vasculature is characterized by an incomplete basement membrane [57].

As discussed above, reduced PDGF signaling alone cannot explain the severity of the *Ccn2−/−* endothelial phenotype. Rather, the data indicate an essential role for CCN2 in formation of the vascular provisional ECM and basement membrane. The relationship between CCN2 and FN expression and function is likely to be complex. CCN2 binds to FN and FN receptors (integrins α4, α5 and β1) [12], [58], [59]. Moreover, loss of CCN2 leads to defective adhesion and spreading of cells on FN, suggesting that these physical interactions are essential for certain cell types, at least *in vitro* [28], [59]. Other studies have shown that CCN2 is required for FN protein and mRNA expression in pathological processes *in vivo* [60], [61]. Studies employing siRNA knockdown approaches demonstrate that CCN2 induces FN expression in various cell types [25], [62]. The studies reported here show that CCN2 induces FN expression in endothelial cells, and that CCN2 is required for normal levels of FN expression during development *in vivo*. While we have focused here on the role of CCN2 as a mediator of FN production by vascular cells, decreased FN synthesis was also seen in fibroblasts in *Ccn2−/−* dermis (Figure 5E,F). These data are consistent with previous studies showing that CCN2 is required for FN synthesis in fibroblasts *in vitro* [61]. Additional studies employing tissue-specific CCN2 knockouts will be required to determine whether the defect in FN synthesis in dermal fibroblasts has physiological consequences.

The reduced deposition of collagen IV in *Ccn2* mutants reveals that CCN2 is an essential regulator of vascular basement membrane formation. The underlying mechanisms by which CCN2 mediates basement membrane formation are unknown. Our studies indicate that CCN2 does not directly regulate levels of expression of *Col4α2*. Therefore, the loss of collagen IV expression in vascular basement membranes may be a secondary consequence of altered FN synthesis and folding. As discussed above, CCN2 directly interacts with FN and its receptors. Increased expression of matrix metalloproteinases (MMPs) that target type IV collagen might also contribute to reduced type IV collagen deposition in endothelial basement membranes. Additional *in vivo* studies will be required to evaluate these possibilities. A growing body of literature implicates CCN2 in abnormal basement membrane thickening in pathological processes. Glomerular basement membrane thickening is prevented in diabetic *Ccn2+/−* mice compared to WT littermates [52]. Moreover, one of the most prominent features in transgenic mice overexpressing CCN2 from the type I collagen promoter is a thickening of endothelial basement membranes [63]. Taken together with the data reported here, CCN2 appears to be a critical mediator of basement membrane formation. CCN2 is required for normal elaboration of the basement membrane during developmental angiogenesis, but CCN2 overexpression leads to basement membrane thickening in multiple fibrotic processes.

The formation of mature endothelial basement membranes involves both pericytes and endothelial cells. While we have focused here on effects of CCN2 in endothelial cells *in vivo*, it is very conceivable that primary defects in both endothelial cells and pericytes in *Ccn2−/−* mice contribute to the basement membrane defects seen in these mutants. It is likely that CCN2 has direct effects on ECM production in pericytes, as CCN2 promotes ECM production and fibroblast activation *in vitro* [64]. Moreover, our preliminary analysis reveals that in addition to the microvasculature, large vessels are impacted by loss of CCN2. This finding raises the possibility that CCN2 plays a direct role in SMCs in addition to pericytes. It is noteworthy that the related matricellular protein CCN1 (Cyr61) is expressed in major vessels, and *Ccn1−/−* mice die early in embryogenesis as a result of defects in large vessel integrity [65]. Although vascular basement membranes have not been investigated in *Ccn1−/−* mice, the defects in vessel integrity raise the possibility that CCN1 and CCN2 will exhibit functional redundancy in vascular elements. It will thus be of interest in future studies to investigate vascular cell recruitment and basement membrane assembly in *Ccn1* and *Ccn1/Ccn2* mutants.

Finally, the use of tissue-specific *Ccn2* knockouts and co-culture experiments will be required to understand the physiological relevance of CCN2 produced by endothelial and mural cells in large vessels.

## Methods

### Ethics Statement

All the experiments related to mice were performed in accordance with National Institutes of Health guidelines for care and use of animals, and also approved by the UCLA Institutional Animal Care and Use Committee (IACUC), protocol #95-018.

### Transgenic Mice

#### *Ccn2−/−* mice

The generation of *Ccn2*−/− mice was described previously [27]. As previously described, *Ccn2+/−* mice appear indistinguishable from WT littermates, and are viable and fertile [27]. *Ccn2−/−* embryos and neonates were obtained by intercrossing *Ccn2+/−* mice. The 4 kb proximal promoter LacZ mice were generated and genotyped as previously described [31]. CCN2-eGFP mice were ordered from the Mutant Mouse Resource Center (MMRC, UC Davis) [32]. All mice were treated and euthanized in accordance with the UCLA Institutional Animal Care and Use Committee (ARC # 1995-018-52A), and the Association of Assessment and Accreditation of Laboratory Animal Care International (AAALAC) guidelines.

#### Histochemical and Immunofluorescent Staining

Freshly isolated embryos were fixed and embedded in paraffin wax as described previously [27]. 5 µm sections were stained with hematoxylin and eosin using standard protocols. LacZ staining was performed as described [66]. Immunofluorescence was performed as described previously [27]. Briefly, paraffin, sections were boiled for 15 min in citrate buffer. Sections were blocked with 5% goat or donkey serum for 1 hour and incubated with primary antibody overnight at 4°C, followed by incubation with secondary antibody for 1 hour at room temperature, then with fluorophore for 30 minutes at room temperature. The following antibodies were used: PECAM (1∶500; MEC 13.3, BD Biosciences), CCN2 (1∶500; L-20 Santa Cruz Biotechnology), NG2 (1∶100; Abcam), Collagen IV (1∶500; Abcam and Santa Cruz Biotech), Desmin (1∶1000; Abcam), anti-Smooth Muscle Actin-FITC (1∶500; Sigma), Col4α2 (1∶1,000; Abcam) and Fibronectin (1∶1,000; Santa Cruz Biotech). Secondary antibodies were conjugated with Alexa-Fluor-555 and Alexa-Fluor-488 (Invitrogen). Sections were counterstained with DAPI (Vectashield). Immunofluoresence was visualized on a Leica TCS-SP Confocal Microscope. For TUNEL staining, the fluorescein In Situ Cell Death Detection Kit (Roche) was used according to manufacturer's protocol. PCNA staining was performed on paraffin sections as described previously [27] using an anti-PCNA antibody (Zymed) and, vessels were identified by PECAM immunofluorescence. The percentage of TUNEL- or PCNA-positive endothelial cells (PECAM-positive) was quantified on digital photomicrographs processed with Photoshop software (Adobe), using Image-Pro software. Pericyte coverage of microvasculature was quantified as described [67]. Capillary density was quantified as the area of PECAM1-positive cells on anti-PECAM1 immunostained images as described [68]. Ten images each for WT and *Ccn2−/−* mice, obtained from 5 independent pairs of littermates, were analyzed. Statistical analysis was performed using Student's *t* test. A *p* value of less than 0.05 was considered statistically significant.

#### Confocal Microscopy

Confocal laser scanning microscopy was performed at the CNSI Advanced Light Microscopy/Spectroscopy Shared Resource Facility at UCLA, supported with funding from NIH-NCRR grant (CJX1-443835-WS-29646) and NSF grant (CHE-0722519). Representative images are shown.

#### Real-time quantitative polymerase chain reaction

RNA was isolated using TRIZOL (Invitrogen) according to the manufacturer's protocol. Synthesis of cDNA was performed with Superscript III (Invitrogen). Semi-quantitative PCR was performed with 20 ng reverse-transcribed RNA. Amplifications were performed for 30 cycles, followed by a 5 min extension at 72°C. Reaction products were gel electrophoreses and quantified using Image Quant software (Molecular Dynamics). Primers for the genes investigated by semi-quantitative RT-PCR were: VegfA and C: VEGFACF 5′-GAA GTC CCA TGA AGT GAT CAA G-3′, VEGF164 5′-CAA GGC TCA CAG TGA TTT TCT GGC-3′; ANG1: ANG1F 5′-CAT TCT TCG CTG CCA TTC TG, ANGR 5′-GCA CAT TGC CCA TGT TGA ATC-3′; PECAM: PECAMF 5′- GAG CCC AAT CAC GTT TCA GTT T-3′, PECAMR 5′-TCC TTC CTG CTT CTT GCT AGC T-3′; Versican0: V_0_F 5′-TTC ACA GAA CGC CAC CCT TGA GTC C-3′, V_0_R 5′-CTA GCT TCT GCA GCT GGC CGG GTC C-3′; Versican1-3: V1F 5′- GCA GCT TGG AGA AAT GGC TTT GAC C-3′, V_1_R 5′- CGA GTA GTT GTG GGT GAT TCC GTG G-3′; PDGFBF 5′-GATCCGCTCCTTTGATGATC-3′, PDGF-BR 5′-GTCTCACACTTGCATGCCAG-3′; PDGFRbetaF 5′-AATGTCTCCAGCACCTTCGT-3′, PDGFRbetaR 5′-AGCGGATGTGGTAAGGCATA-3′ [69]; GAPDH, GapdhF 5′-GCA GTG GCA AAG TGG AGA TT-3′; GapdhR 5′-AGT GGA TGC AGG GAT GAT GT. cDNA was amplified using Sybr Green I PCR Master Mix (Applied Biosystems). Amplicons were generated and analyzed with the ABI 7000 Real-time PCR system (Applied Biosystems). Data were normalized to the levels of *Gapdh*. Triplicate assays were run and analyses were repeated three times. Specificity was tested by measurement of Tm-values and by gel electrophoresis of the amplicons. Data are represented as the means of relative levels of expression+the S.E. of the mean, and statistical analysis was performed with Student's *t* test. A *p* value of less than 0.05 was considered statistically significant.

#### Flow Cytometry

FACS analysis was performed as previously described [70]. Brain, liver and lung samples were harvested from E16.5 CCN2 wild type and mutant embryos. Single cell suspensions were created by serial syringe digestion in 0.2% Collagenase (Sigma *Clostridium histolyticum* C2674-6), 0.05% Dispase (Invitrogen 17105-041), 0.0075% DnaseI (Sigma D4513), 0.02% Penicillin Streptomycin (GIBCO-Invitrogen 15140148) in 1× PBS/10%Fetal Bovine Serum (GIBCO-Invitrogen 10437-028). Cell suspensions were incubated with the following primary antibodies: CD45-APC Cy7 (1∶200;Abcam); NG2 (1∶200; Abcam); CD31-PE (1∶200; Abcam); PDGFRβ-APC (1∶50; Invitrogen). A secondary goat anti-rabbit conjugated antibody 488 (Invitrogen) was used for the unconjugated NG2 antibody. FITC, APC, APC-Cy7, PE control beads (Invitrogen) and 488 secondary alone were used as controls to correct for background florescence and gate parameters. FACS sorting was performed using the LSRII FACS analyzer and cell counts were plotting by FlowJo analysis (TreeStar).

#### Transmission Electron Microscopy

Ultrastructural analysis was performed on dermal microvasculature by the University of California, Los Angeles, Electron Microscopy Core Facility. 10 images were taken from each E18.5 embryo. Four *Ccn2−/−* and four WT littermates were examined. Representative images are shown.

#### Cell lines and treatments

Human umbilical vein endothelial cells (HUVECs a gift from Dr. Jau-Nian Chen) were maintained in HUVEC culture media (Sigma) as described previously [71]. HUVECs were maintained in 0.5% serum for 12 hr prior to treatment with recombinant protein. Cells were treated with 150 ng/ml recombinant (r) CCN2 (Peprotech) and/or 150 ng/ml rPDGF-B (Peprotech), using serum free treated cells as control. Mouse vascular smooth muscle (MOVAS) (ATTC) cells were cultured in DMEM, 10% FBS. MOVAS cells were washed with Hepes buffered saline (HBS) containing 5 mM MgCl_2_ (HBS+Mg), and treated with or without 150 ng/ml rPDGF-B in DMEM, 0.5% FBS for the indicated times. In other experiments, MOVAS cells were transiently transfected with pcDNA3-CCN2-HA [72] using Lipofectamine (Invitrogen), and treated with 150 ng/ml rPDGF-B 24 hrs later for the indicated time periods. Each experiment was performed in triplicate and repeated at least twice. HUVECs were also transfected with CCN2-GFP adenovirus and adenoviral control vectors at a multiplicity of infection (MOI) of 200 (a kind gift of Dr. Fayez Safadi).

#### Western blot analysis

 Cells were lysed with RIPA buffer with 1× protease (Complete Mini Roche) and 1× phosphatase inhibitors (Cocktail 2, Sigma). Lysates were separated by 6–12% SDS-PAGE and transferred to nitrocellulose membrane (0.45 um; BioRad). Membranes were incubated with antibodies against CCN2 (L-20; 1∶2,000, Santa Cruz Biotechnology), PDGF-B (1∶2000, Cell Signaling), PDGFR β (1∶2,000 Cell Signaling), STAT3 (1∶1,000, Cell Signaling), pSTAT3 (1∶2,000, Cell Signaling), total AKT (1∶2,000, Cell Signaling), phospho-AKT (1∶2000, Cell Signaling), phospho-ERK1/2 (1∶2,000, Cell Signaling), Collagen type IV (1∶2,000; Abcam), Fibronectin (1∶2,000; Santa Cruz Biotech) and actin (1∶5,000, Sigma). Antibody-antigen complexes were detected with HRP-conjugated secondary goat and rabbit antibodies (Bio-Rad). Western blots were performed in triplicate and normalized to actin. Quantification was performed using ImageJ. Statistical analysis was performed using the Student's t-Test, and a p-value less than 0.05 was considered significant. Representative western blots are shown.

## Supporting Information

Methods S1 Methods for co-immunoprecipitation and western blot analysis (Figure S5).
(DOCX)

Figure S1 **Expression of CCN2 in vasculature and vascular defects in** ***Ccn2*** **mutants.** (A) Confocal image of dermal microvasculature immunostained for CCN2 (green) and PECAM (red). Yellow indicates co-expression in endothelial cells. The staining is punctate, as reported previously [30]. Associated mural cells expressing CCN2 (green) are indicated by arrows. Endothelium demonstrating CCN2 expression is indicated by arrowheads. (B,C) Confocal images of fetal placenta from E16.5 WT (B) and *Ccn2−/−* (C) littermates immunostained for NG2 (green) and PECAM (red) and counterstained with DAPI showing no obvious changes in vascular organization. (D) E14.5 WT and (E) *Ccn2−/−* littermate. Arrows highlight dilation of cerebral vessels in the mutant. Dilated vessels are apparent in the mutant. (F–I) Confocal images of immunofluorescence staining for αSMA (green) and PECAM (red) in dorsal dermis of newborn (P0) WT (F,H,) and *Ccn2−/−* (G,I,) littermates. Arrows in (F–I) indicate arteries; arrowheads demarcate veins. (J,K) Confocal images of immunofluorescence staining for αSMA (green) and PECAM (red) in dorsal dermis of newborn (P0) WT (J) and *Ccn2−/−* (K) littermates showing paired arterioles (arrows) and venules (arrowheads). (L,M) Confocal images of immunofluorescence staining for EphB4 (green) and PECAM (red) of E16.5 WT (L) and Ccn2−/− littermate (M) dorsal dermal microvasculature.
(TIF)

Figure S2 **Altered gene expression in Ccn2 mutants.** (A) Quantification of microvessel density. (B,C) Additional representative confocal images of PECAM-immunostained dorsal dermal microvasculature from WT (B) and *Ccn2−/−* (C) E18.5 littermates showing increased vessel density in mutants. (D) Representative image of paraffin section through E16.5 dorsal dermis analyzed by αPECAM and αPCNA co-immunofluorescence and counterstained with DAPI, used to assess endothelial cell proliferation. Image from WT dermis is shown. Arrows point to PCNA-positive endothelial cells. (E) Quantification of PCNA-positive cells revealed no differences in proliferation in WT versus mutant vessels. (F) Representative images of paraffin section through E16.5 dorsal dermis analyzed by immunostaining for PECAM and TUNEL-positive endothelial cells and counterstained with DAPI. Image from WT dermis is shown. (G) Quantification of TUNEL-positive endothelial cells revealed no evidence for altered levels of cell death in *Ccn2* mutant vasculature. (H–K) Quantitative RT-PCR analysis of relative levels of expression of (H) *Ang1*, (I) *Vegf164*, (J) *Versican1*, and (K) *Versican0* mRNA in WT and *Ccn2−/−* E16.5 vasculature. *, p<0.05.
(TIF)

Figure S3 **FACS analysis of pericyte or endothelial cell number in** ***Ccn2*** **mutants.** (A, C) FACS analysis of (A) WT and (C) *Ccn2−/−* skin samples analyzed for expression of PDGFRβ. (B, D) FACS analysis of (B) WT and (D) *Ccn2−/−* skin samples analyzed for expression of NG2. (E) Quantification of percentages of PDGFRb, NG2, and PECAM-expressing cells revealed no differences.
(TIF)

Figure S4 **Defective pericyte association with endothelium in** ***Ccn2*** **mutants.** Paraffin sections through E16.5 dermis immunostained with desmin (red) and counterstained with DAPI. (A,B) WT desmin positive pericytes appear elongated and cover most of the surface of the microvessels. (C,D) *Ccn2−/−* desmin-positive pericytes have a rounder appearance and desmin staining has a less uniform appearance.
(TIF)

Figure S5 **No physical interaction between CCN2 and PDGF-B or PDGFRβ.** (A) No physical interactions between CCN2 and PDGF-B. MOVAS cells were infected with a lentiviral vector encoding CCN-HA (M-CCN2 cells). Non-crosslinked or DSP-crosslinked lystaes (see Supplementary Materials and Methods) were immunoprecipitated with αHA antibody. Western blots of the immunoprecipitates were probed with αCCN2 and αPDGFB antibodies. First lane in each panel shows rCCN2 and rPDGFB standards. TXsol and TX insol, triton X-soluble and –insoluble pellets, respectively. (B) No direct interactions between CCN2 and PDGFRβ. M-CCN2 cells were treated with or without PDGF-B, followed by immunoprecipitation with αHA antibody. Western blots of the immunoprecipitates were probed with αPDGFRβ (PDGFR) or αphospho (Y751) PDGFRβ antibody.
(TIF)

Figure S6 **CCN2 induces fibronectin expression in endothelial cells.** Quantification of relative levels of expression of fibronectin (FN) and Col IV in endothelial cells in the presence or absence of CCN2. See legend to Figure 5 for experimental details. Induction of FN was seen by 8 hrs. There was a trend towards increased FN at 12 hrs (p<0.06), but this did not reach statistical significance. *, p<0.05. There was no significant increase in Col IV levels at any time point.
(TIF)
