# Supplementary material for: CCN2/Connective Tissue Growth Factor Is Essential for Pericyte Adhesion and Endothelial Basement Membrane Formation during Angiogenesis
Source: PLoS One. 2012 Feb 20;7(2):e30562. doi: 10.1371/journal.pone.0030562 (PMC3282727; doi:10.1371/journal.pone.0030562)
Supplement: Methods S1 — Methods for co-immunoprecipitation and western blot analysis (Figure S5). (DOCX) [file pone.0030562.s001.docx]

**Methods**

**Co-Immunoprecipitation Assays and Western blot analysis.** For analysis of physical interactions between CCN2 and PDGF, the M-CCN2 cell line was derived from MOVAS cells infected with a lentiviral construct encoding HA-tagged CCN2 regulated by a CMV promoter (OPEN Biosystems). Cells were selected and cultured in DMEM, 10% FBS with 4 ng/mL puromycin. Immunoprecipitations were performed by incubation of M-CCN2 cells with or without 45ng rPDGF for 5 minutes. Crosslinking was performed by rocking cells for 15 minutes in HBS+Mg containing DSP (Dithiobis, Pierce). DSP solution was replaced with ice-cold HBS+Mg containing 50 mM ammonium chloride, pH 7.5 for 10 minutes. Cells were lysed with lysis buffer containing 50 mM NH_4_Cl. The TritonX 100-insoluble pellet was resuspended in non-reducing sample buffer. Insoluble material was removed by centrifugation. 4 volumes of acetone were added to the supernatant and held overnight at -80°C. Precipitated crosslinked proteins were dissolved in 0.25% SDS in HBS. The protein solution was denatured by boiling and sonication. Lysates were immunoprecipitated with Protein-G magnetic beads (Millipore) displaying HA antibody (Bethyl). Antibody beads without exposure to lysates were used as a negative control. rPDGF and whole cell lysates of M-CCN2 cells were used as standards for rPDGF and CCN2, respectively. Cells were lysed in HBS, 5mM MgCl_2_, 1% Triton X-100, 1% Protease (Roche), phosphatase inhibitors (Sigma) for 20 min with rotation at 4°C. In some experiments, the pellet was re-extracted with Triton X to generate TX and TX-insoluble pellets. Lysates were separated by 10% SDS-PAGE and transferred to nitrocellulose membrane (BioRad). Membranes were incubated with antibodies against CCN2 (L-20 Santa Cruz Biotechnology), PDGF-B (1:2000, Cell Signaling), PDGFR β (1:2,000 Cell Signaling). Experiments were performed in triplicate. Representative blots are shown.
