# Supplementary figures and images for: CCN2/Connective Tissue Growth Factor Is Essential for Pericyte Adhesion and Endothelial Basement Membrane Formation during Angiogenesis
Source: PLoS One. 2012 Feb 20;7(2):e30562. doi: 10.1371/journal.pone.0030562 (PMC3282727; doi:10.1371/journal.pone.0030562)

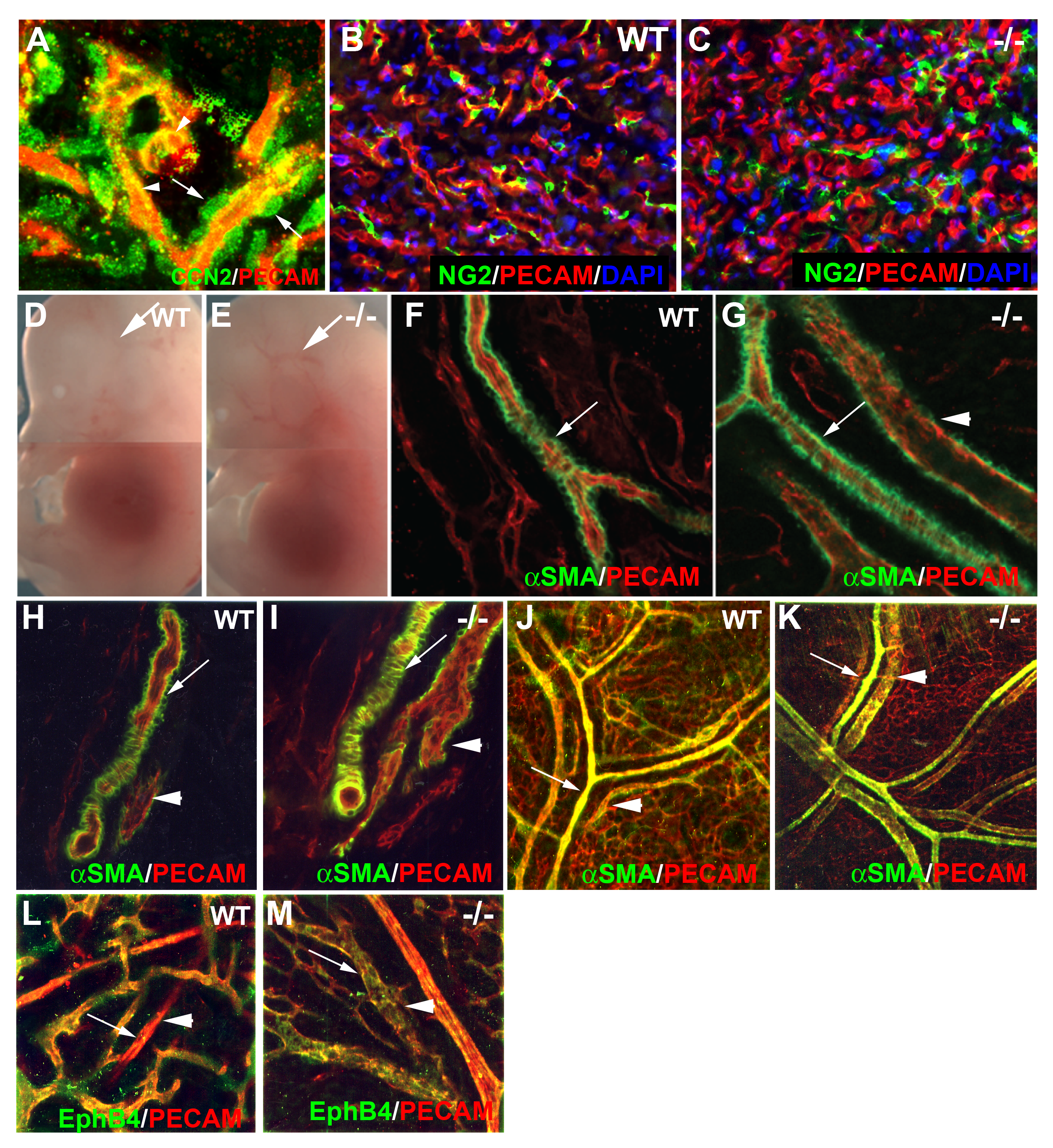

Supplement: Figure S1 — Expression of CCN2 in vasculature and vascular defects in Ccn2 mutants. (A) Confocal image of dermal microvasculature immunostained for CCN2 (green) and PECAM (red). Yellow indicates co-expression in endothelial cells. The staining is punctate, as reported previously [30]. Associated mural cells expressing CCN2 (green) are indicated by arrows. Endothelium demonstrating CCN2 expression is indicated by arrowheads. (B,C) Confocal images of fetal placenta from E16.5 WT (B) and Ccn2−/− (C) littermates immunostained for NG2 (green) and PECAM (red) and counterstained with DAPI showing no obvious changes in vascular organization. (D) E14.5 WT and (E) Ccn2−/− littermate. Arrows highlight dilation of cerebral vessels in the mutant. Dilated vessels are apparent in the mutant. (F–I) Confocal images of immunofluorescence staining for αSMA (green) and PECAM (red) in dorsal dermis of newborn (P0) WT (F,H,) and Ccn2−/− (G,I,) littermates. Arrows in (F–I) indicate arteries; arrowheads demarcate veins. (J,K) Confocal images of immunofluorescence staining for αSMA (green) and PECAM (red) in dorsal dermis of newborn (P0) WT (J) and Ccn2−/− (K) littermates showing paired arterioles (arrows) and venules (arrowheads). (L,M) Confocal images of immunofluorescence staining for EphB4 (green) and PECAM (red) of E16.5 WT (L) and Ccn2−/− littermate (M) dorsal dermal microvasculature. (TIF) [file pone.0030562.s002.tif]

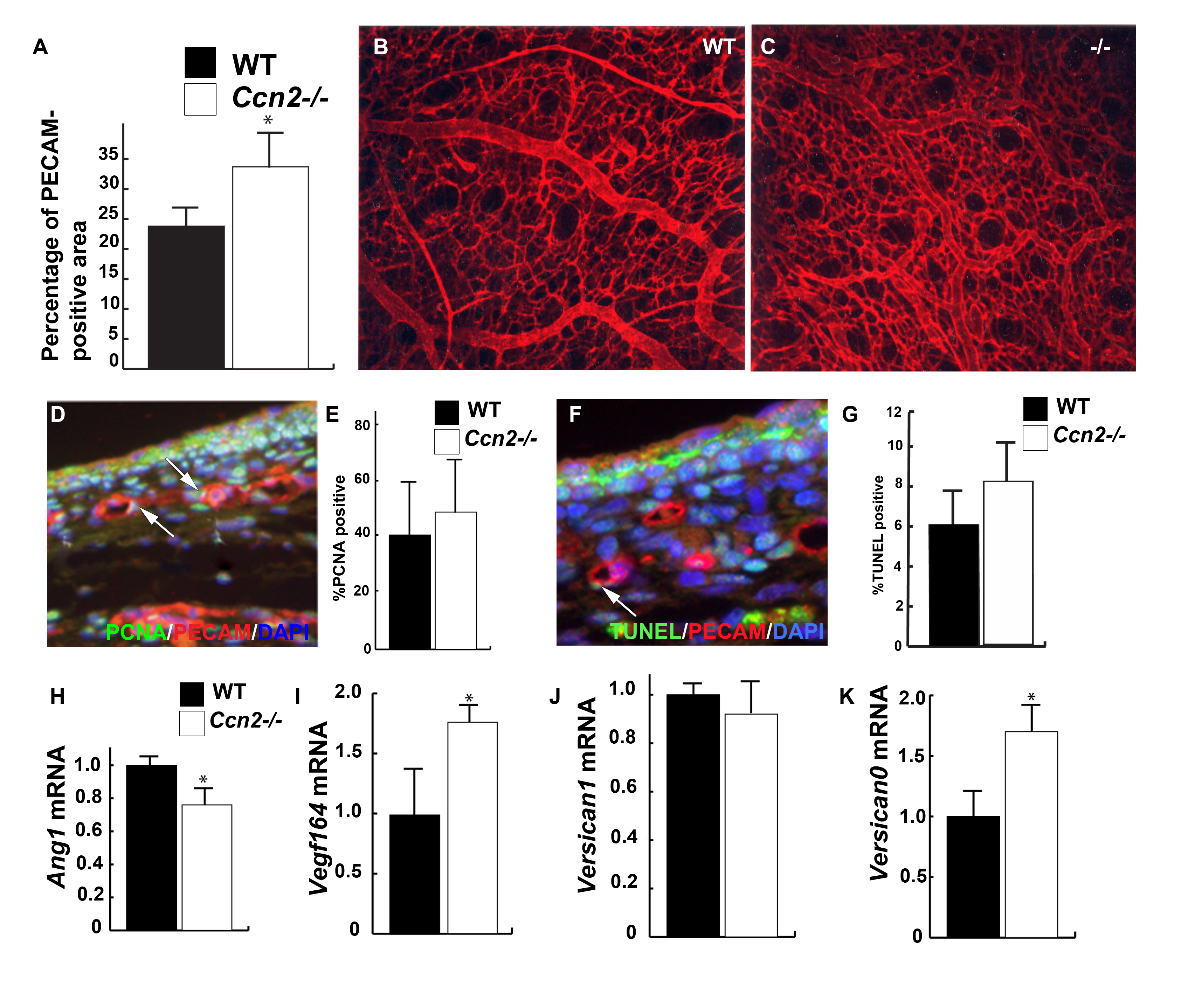

Supplement: Figure S2 — Altered gene expression in Ccn2 mutants. (A) Quantification of microvessel density. (B,C) Additional representative confocal images of PECAM-immunostained dorsal dermal microvasculature from WT (B) and Ccn2−/− (C) E18.5 littermates showing increased vessel density in mutants. (D) Representative image of paraffin section through E16.5 dorsal dermis analyzed by αPECAM and αPCNA co-immunofluorescence and counterstained with DAPI, used to assess endothelial cell proliferation. Image from WT dermis is shown. Arrows point to PCNA-positive endothelial cells. (E) Quantification of PCNA-positive cells revealed no differences in proliferation in WT versus mutant vessels. (F) Representative images of paraffin section through E16.5 dorsal dermis analyzed by immunostaining for PECAM and TUNEL-positive endothelial cells and counterstained with DAPI. Image from WT dermis is shown. (G) Quantification of TUNEL-positive endothelial cells revealed no evidence for altered levels of cell death in Ccn2 mutant vasculature. (H–K) Quantitative RT-PCR analysis of relative levels of expression of (H) Ang1, (I) Vegf164, (J) Versican1, and (K) Versican0 mRNA in WT and Ccn2−/− E16.5 vasculature. *, p<0.05. (TIF) [file pone.0030562.s003.tif]

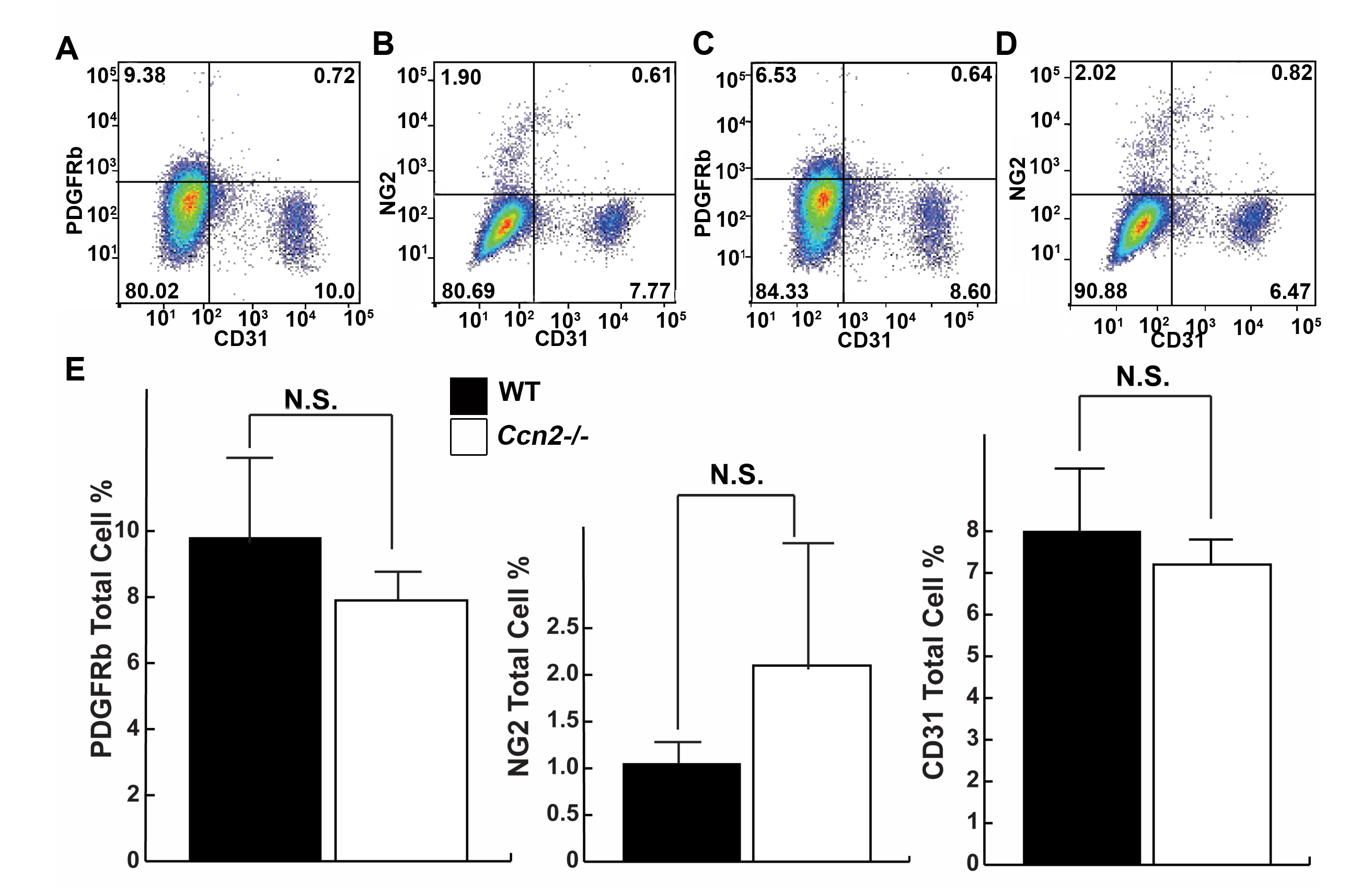

Supplement: Figure S3 — FACS analysis of pericyte or endothelial cell number in Ccn2 mutants. (A, C) FACS analysis of (A) WT and (C) Ccn2−/− skin samples analyzed for expression of PDGFRβ. (B, D) FACS analysis of (B) WT and (D) Ccn2−/− skin samples analyzed for expression of NG2. (E) Quantification of percentages of PDGFRb, NG2, and PECAM-expressing cells revealed no differences. (TIF) [file pone.0030562.s004.tif]

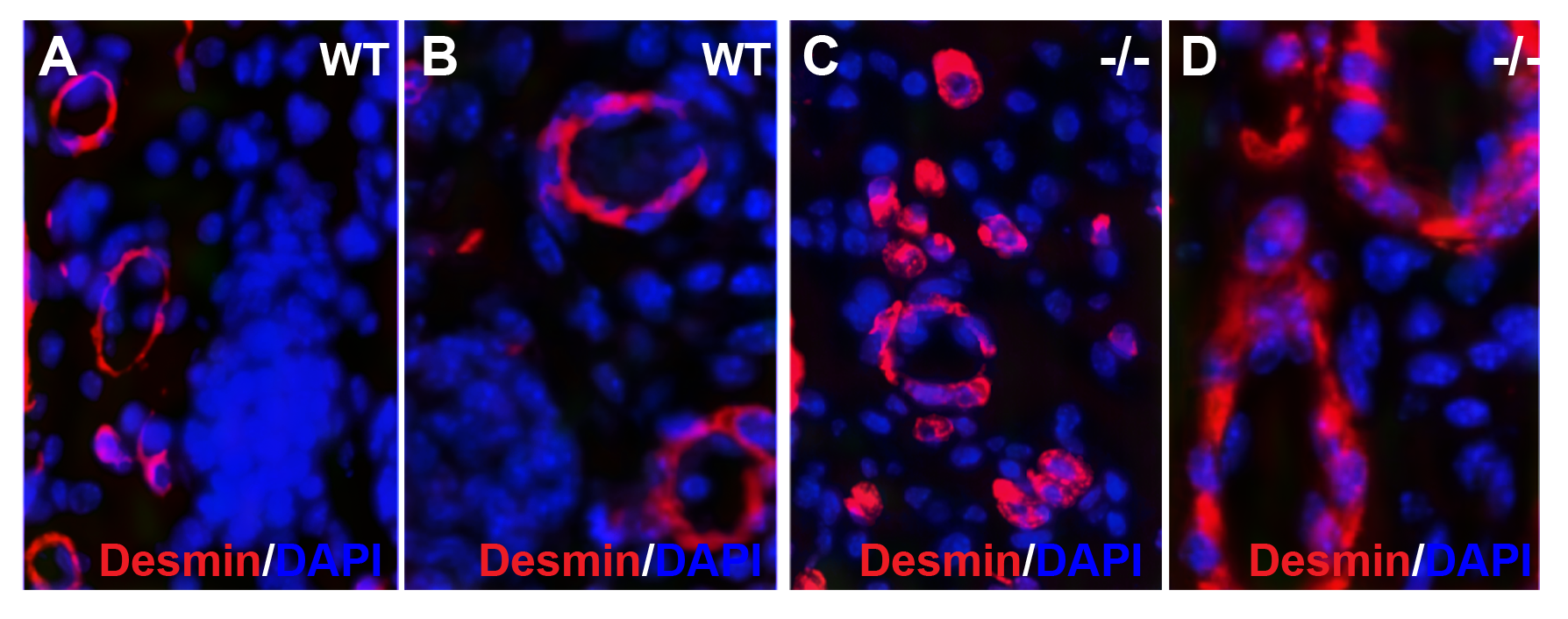

Supplement: Figure S4 — Defective pericyte association with endothelium in Ccn2 mutants. Paraffin sections through E16.5 dermis immunostained with desmin (red) and counterstained with DAPI. (A,B) WT desmin positive pericytes appear elongated and cover most of the surface of the microvessels. (C,D) Ccn2−/− desmin-positive pericytes have a rounder appearance and desmin staining has a less uniform appearance. (TIF) [file pone.0030562.s005.tif]

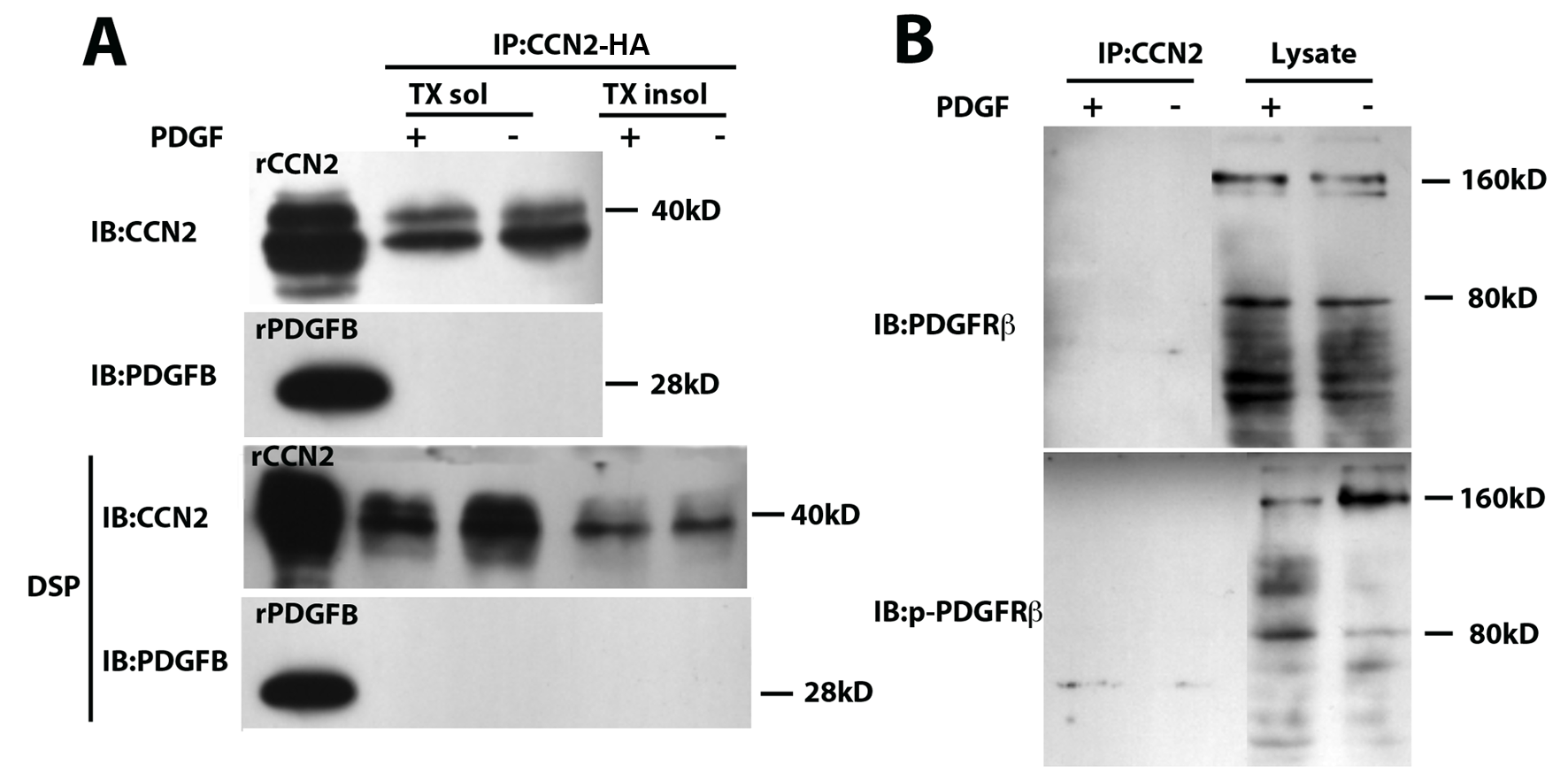

Supplement: Figure S5 — No physical interaction between CCN2 and PDGF-B or PDGFRβ. (A) No physical interactions between CCN2 and PDGF-B. MOVAS cells were infected with a lentiviral vector encoding CCN-HA (M-CCN2 cells). Non-crosslinked or DSP-crosslinked lystaes (see Supplementary Materials and Methods) were immunoprecipitated with αHA antibody. Western blots of the immunoprecipitates were probed with αCCN2 and αPDGFB antibodies. First lane in each panel shows rCCN2 and rPDGFB standards. TXsol and TX insol, triton X-soluble and –insoluble pellets, respectively. (B) No direct interactions between CCN2 and PDGFRβ. M-CCN2 cells were treated with or without PDGF-B, followed by immunoprecipitation with αHA antibody. Western blots of the immunoprecipitates were probed with αPDGFRβ (PDGFR) or αphospho (Y751) PDGFRβ antibody. (TIF) [file pone.0030562.s006.tif]

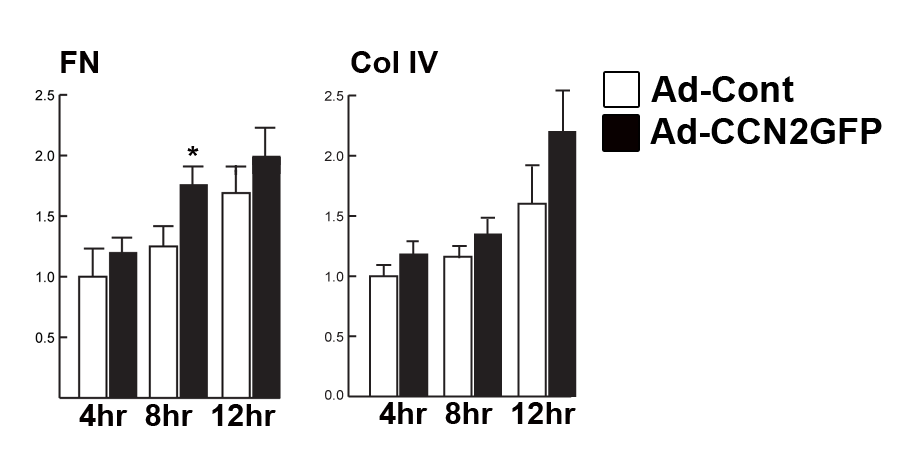

Supplement: Figure S6 — CCN2 induces fibronectin expression in endothelial cells. Quantification of relative levels of expression of fibronectin (FN) and Col IV in endothelial cells in the presence or absence of CCN2. See legend to Figure 5 for experimental details. Induction of FN was seen by 8 hrs. There was a trend towards increased FN at 12 hrs (p<0.06), but this did not reach statistical significance. *, p<0.05. There was no significant increase in Col IV levels at any time point. (TIF) [file pone.0030562.s007.tif]
